# Editorial: Children's health and screen time

**DOI:** 10.3389/fpubh.2025.1769843

**Published:** 2026-01-19

**Authors:** Dandan Wu, Georgios Floros, Hui Li

**Affiliations:** 1 Department of Early Childhood Education, Faculty of Education and Human Development, The Education University of Hong Kong, Hong Kong, Hong Kong SAR, China; 2 AI, Brain and Child Research Centre, Academy for Educational Development and Innovation, The Education University of Hong Kong, Hong Kong, Hong Kong SAR, China; 3 Centre for Psychosocial Health, Faculty of Education and Human Development, The Education University of Hong Kong, Hong Kong, Hong Kong SAR, China; 4 2nd Department of Psychiatry, Medical School, Aristotle University of Thessaloniki, Thessaloniki, Greece; 5 Macquarie School of Education, Macquarie University, Sydney, NSW, Australia

**Keywords:** adolescent wellbeing, child health, digital media, methodology, parental mediation, screen time

The lives of children and adolescents today are inextricably woven into the fabric of the digital world. From the first touch of a tablet in toddlerhood to the constant social hum of a smartphone in the teenage years, screens have become ubiquitous. This digital immersion offers unprecedented opportunities for learning and connection, yet it simultaneously presents a complex web of challenges. The question is no longer *if* children will use screens, but *how* this use shapes their wellbeing.

This special Research Topic brings together 20 empirical studies, reviews, and methodological papers to advance this urgent conversation. It does not claim to offer final answers in a field where technology outpaces research. Rather, it aims to provide a nuanced, evidence-based exploration that moves beyond simplistic dichotomies of “good” vs. “bad” screen time. By grappling with the intricate mechanisms, contextual factors, and intervention strategies, this collection marks a significant step toward a more sophisticated understanding of the relationship between screen use and child health.

## A thematic journey through a contested landscape

The proliferation of digital screens has fundamentally reshaped childhood. This special issue, “Children's Health and Screen Time,” assembles 20 diverse articles that dissect the multifaceted impacts of digital engagement by examining content, context, and the developing child. To navigate this rich and at times contested body of research, we have organized the contributions into four key themes: (1) The Core Impacts on Physical, Mental, and Social Development; (2) The Critical Role of Context: Family and Culture; (3) From Risk to Resilience: Problematic Use and Protective Factors; and (4) Confronting Methodological Hurdles and Sharpening Our Tools.

## Theme 1: the core impacts on physical, mental, and social development

This collection first reaffirms and deepens our knowledge of the foundational, though often correlational, links between screen use and key health outcomes. This knowledge is increasingly grounded in neuroscience; a recent scoping review by Wu et al. (1) synthesized 33 neuroimaging studies and concluded that digital experience induces both positive and negative structural and functional changes in the developing brain, identifying the prefrontal cortex—the neural basis of executive function—as a particularly vulnerable area. The studies in this issue provide granular evidence of these impacts. At the most acute end of the mental health spectrum, the meta-analysis by He et al. provides sobering evidence of a significant association between Problematic Internet Use (PIU) and suicidal behaviors in Chinese adolescents. Broadening the scope, Martínez-Iniesta et al. find that addictive social network behaviors are linked to lower self-esteem.

Sleep consistently emerges as a critical process disrupted by screens, as demonstrated by Yang and Shanshan, who show entertainment screen time negatively impacts sleep quality in both Chinese and British adolescents. For younger children, concerns center on development. The systematic review by Misirli et al. stresses that the impact of touchscreen exposure on social functioning is not uniform but shaped by content and adult mediation. Elaborating on this, Regehr et al. demonstrate how harmful content is increasingly driven into the online content that young people consume by social media algorithms, with profound implications for the normalization of hate, misogyny and racism. Complicating a simple cause-and-effect narrative, the longitudinal study by Soto-Ramirez et al. reveals a bidirectional relationship, where early externalizing problems in toddlers can predict higher screen use later on, suggesting that screens can be both a cause and a consequence of developmental challenges. Finally, linking these threads, studies by Feng et al. and Qu et al. demonstrate how screen time's negative correlation with academic performance and learning quality is channeled through a cascade of mediating factors like impaired executive function, poor sleep, and mental health.

## Theme 2: the critical role of context: family, culture, and content

The impact of screen time is not deterministic; it is profoundly shaped by a child's environment, challenging the efficacy of one-size-fits-all prescriptions. This extends beyond just the family or culture to the very nature of the digital activity itself. Reinforcing this issue's central thesis, a large-scale study by Cao and Li (2) found that for preschoolers, the *type* of digital engagement—not the total duration—was the key predictor of their well-being profile. Entertainment-focused activities were associated with higher-risk profiles, whereas creative activities acted as a protective factor. This crucial distinction between content and mere time underscores the need to look inside the “black box” of screen use. Adverse events during this sensitive period may leave a mark in the future, as a study by Elkin et al. showed that university students with childhood trauma had higher social media addiction.

Several articles in this issue further underscore the pivotal role of the family. Zhang reveals that harsh parenting can indirectly increase screen time, while Chen et al. find that active, supportive parental mediation is more protective than simple supervision. For families of children with Autism Spectrum Disorder, Ebrahim et al. highlight the need for highly tailored strategies, moving beyond simple restriction to conditional access and preparation for cessation.

Expanding the lens, comparative studies reveal that global trends manifest with local nuances. Yang and Shanshan find that culture moderates the pathway from screen time to self-control, with a stronger negative impact among British youth compared to Chinese youth. Similarly, Kornienko et al. find that while higher screen time is universally linked to more health problems in Russia and Cuba, the *specific* issues that arise differ based on socio-cultural context.

## Theme 3: from risk to resilience: problematic use, protective factors, and interventions

Moving from identifying problems to exploring solutions, this theme highlights a crucial tension between clinical frameworks and lived experience. The qualitative study by Bore et al. offers a patient-centered perspective on gaming disorder treatment, finding that adolescents value a holistic approach that addresses underlying life difficulties, rather than framing gaming as the sole problem. This resonates with the review by Di Iorio et al., which argues for assessing social media use non-judgmentally as an integral part of understanding a young person's social world.

In a proactive shift, Martin-Barrado and Gomez-Baya show how the Positive Youth Development (PYD) framework acts as a protective factor against digital harms. Furthermore, Zhang et al. (2025) provide experimental evidence that game design itself can be leveraged for positive outcomes, as playing a cooperative game significantly increased sharing behavior in young children. These studies suggest that the most effective interventions may not be about eliminating screens, but about building internal resilience and redesigning the digital environment itself.

## Theme 4: confronting methodological hurdles and sharpening our tools

Underpinning all progress is the refinement of our research methods. For too long, the field has been hampered by crude metrics like parent-reported screen hours, which fail to capture the nature of engagement, and a lack of tools validated for our youngest digital natives. This special issue features two articles that directly confront these challenges. Thompson et al. address a key gap by developing and validating the “Affinity-TV” and “Affinity-Mobile” scales, moving beyond simple duration to measure a toddler's *attraction* to screen devices as a potential early risk indicator. Similarly, Song et al. contribute a robust parent-report tool for Chinese preschoolers (MBQ-C) that integrates the measurement of physical activity, screen time, and sleep. These methodological advances are not mere technicalities; they are essential for building a more accurate and defensible evidence base in a rapidly evolving field.

## Conclusion: toward a more sophisticated roadmap

The articles in this special issue, taken together, paint a picture of great complexity. A unifying thread is the move from asking *if* screen time is harmful to exploring *how, why*, and *for whom*. The evidence illuminates a series of intricate mechanisms: we see how screen time can erode internal resources like self-control (Yang and Shanshan), how physical health (BMI) can mediate its effects on mental health, and how good sleep can act as a protective buffer (Wang et al.).

While this collection marks a significant step forward, it is by no means the final word. Critical questions regarding the long-term causal impacts of screen use, the influence of emerging AI-driven content algorithms, and the socioeconomic disparities in digital access and literacy remain urgent areas for future inquiry. The heavy reliance on correlational data across the field still makes definitive causal claims challenging.

Nevertheless, the evidence presented here provides a clear mandate. We must shift our focus from mere restriction to fostering mindful and intentional digital citizenship. This involves promoting active parental mediation, strengthening children's internal self-regulation, tailoring strategies to developmental and cultural contexts, and demanding high-quality, prosocial content from industry. The digital world is here to stay; this research provides an invaluable, albeit incomplete, roadmap to help us guide the next generation in navigating its maze—not just to survive, but to thrive.

## References

[1] Wu D Dong X Liu D Li H. How early digital experience shapes young brains during 0–12 years: a scoping review. Early Educ Dev. (2024) 35:1395–431. doi: 10.1080/10409289.2023.2278117

[2] Cao S Li H. Digital activities, not screen time, associated with preschoolers' well-being: evidence from china. Early Child Educ J. (2025). doi: 10.1007/s10643-025-01992-x

